# The impact of sample imbalance on identifying differentially expressed genes

**DOI:** 10.1186/1471-2105-7-S4-S8

**Published:** 2006-12-12

**Authors:** Kun Yang, Jianzhong Li, Hong Gao

**Affiliations:** 1 Department of Computer Science and Engineering, Harbin Institute of Technology, Harbin, 150001, China

## Abstract

**Background:**

Recently several statistical methods have been proposed to identify genes with differential expression between two conditions. However, very few studies consider the problem of sample imbalance and there is no study to investigate the impact of sample imbalance on identifying differential expression genes. In addition, it is not clear which method is more suitable for the unbalanced data.

**Results:**

Based on random sampling, two evaluation models are proposed to investigate the impact of sample imbalance on identifying differential expression genes. Using the proposed evaluation models, the performances of six famous methods are compared on the unbalanced data. The experimental results indicate that the sample imbalance has a great influence on selecting differential expression genes. Furthermore, different methods have very different performances on the unbalanced data. Among the six methods, the welch t-test appears to perform best when the size of samples in the large variance group is larger than that in the small one, while the Regularized t-test and SAM outperform others on the unbalanced data in other cases.

**Conclusion:**

Two proposed evaluation models are effective and sample imbalance should be taken into account in microarray experiment design and gene expression data analysis. The results and two proposed evaluation models can provide some help in selecting suitable method to process the unbalanced data.

## Background

Microarrays enable us to monitor expressions of thousands of genes simultaneously and generate enormous amount of data. Using such techniques, it is possible to explore the secret of biology at the molecular level and understand the fundamental biological processes ranging from gene function to development and to cancer [1-3]. In microarray experiments, the expression levels of several thousands candidate genes have been monitored in two opposite conditions, such as Treatment versus Control conditions, where each condition is represented by several samples. Unfortunately, most monitored genes are unrelated to the conditions and their expression levels do not change or change by chance, while other genes are strongly related to the conditions and truly change their expression levels according to conditions. However, these differentially expressed genes are very useful in latter research and clinical applications [2,3]. Therefore, one of the important tasks in microarray data analysis is to compare the expression levels of genes in samples drawn from two different conditions and to select genes with differential expression under those two conditions. Specifically, we are interesting in identifying which of several thousands candidate genes have had their expression levels changed by condition, given a microarray data.

One simple approach used in literature to detect differential expression genes is "fold change" method, in which a gene is declared to be differentially expressed if its average expression level varies by more than a given constant between two conditions. However, "fold change" method has been demonstrated to be unreliable and inefficient, because statistical variability is not considered [4]. Then, many sophisticated statistical approaches have been proposed [5,6]. These approaches can be roughly classified into two categories. The parametric methods based on statistical model is the first category of methods. This kind of methods include various versions of the two-sample t-test [6-8]. Due to the reason that gene expression data are often noisy and not normally distributed [9], the strong assumption of parametric method can be violated in practice. The second category of approaches is nonparametric statistical methods, including the Wilcoxon rank-sum test [10], the Significance Analysis of Microarray (SAM) method [11], the Empirical Bayes (EB) method [12], the mixture model method [13] and other modified nonparametric methods [14,15]. For recent reviews, please see [5,6].

However, very few studies consider the problem of sample imbalance in detecting differential expression genes and there are no studies as well as quantitive method to investigate the effect of sample imbalance on differential expression genes selection. Sample imbalance means that the size of samples in one group is very different to that in another group. In fact, the problem of sample imbalance usually appears in gene expression data, especially in the data about tumor samples. For example, the data in [16-23] are all unbalanced. There are many factors causing the problem of sample imbalance, such as the limit of source of tumor samples, budgetary constraints and reducing samples in the control group artificially and factitiously. Coupled with the small sample in gene expression data, the problem of sample imbalance may be more serious. Consequently, two important and natural questions may be asked by biologists as follows: How does the sample imbalance affect the methods for identifying differential expression genes? Which method is more suitable for the unbalanced data? In addition, previous studies [24,25] have found that the variability of gene expression may be related to the average expression. It suggests that the two sample t-test being used should be based on unequal variances. An instant but reasonable question is: whether the above suggestion is still true on the unbalanced data.

In this paper, we investigate the new problem about the impact of sample imbalance on identifying differential expression genes. Two evaluation models based on random sampling are proposed and six famous methods are compared on both the real data and the simulated data. Under each evaluation model, the random sampling is utilized to estimate the expected performances of methods on the unbalanced data which satisfy one specific sample ratio between two groups. Then the variations of performances are used to illustrate the effect of sample imbalance on differential expression genes selection and method selection.

## Results

In this section, six methods including two-sample t-test with equal variances (equalling F-test) [6], two-sample t-test with unequal variances (i.e. Welch t-test) [5,7], Wilcoxon rank-sum test [10], SAM [11], Regularized t-test [8] and the permutation-based method of Pan [15] are systematically compared on real data and simulated data according to two evaluation models. All experiments are conducted in Matlab environment on a Pentium PC with a 3.20 GHz CPU and 512 MB RAM. The processing procedure is as follows. For every pair of fixed parameters *n*_1 _and *n*_2 _(which are the numbers of samples in class one *C*_1 _and class two *C*_2_) in each experiment under two evaluation models, first, we randomly create a set of *x *independent artificial data or simulated data and test all six methods on these *x *data to get the results. For a specific method, each one in the *x *random data will only get one result for each measure, for example Overlap Rate, Precision Rate or Recall Rate. Then, these *x *values are treated as a random sample of size *x *from the fixed parameters *n*_1 _and *n*_2_. Last, the expected performance of each method and its 0.95 confidence interval are calculated from this kind of random samples.

### Datasets

Two real datasets are the liver dataset [21] and the prostate dataset [26]. Taking a data preprocess protocol similar to that in Dudoit et al [27], we screen out genes with missing data in more than 5% arrays, impute other missing data by 0, and then apply a base 2 logarithmic transformation. Each experiment is standardized to zero median across the genes. The prostate data finally consists of gene expression profiles of 62 primary prostate tumours and 41 normal specimens with expression values of 7931 genes. The liver data consists of gene expression profiles of 105 primary HCC and 76 non-tumor liver tissues, 7 benign liver tumor samples, 10 metastatic cancers, and 10 HCC cell lines on 11763 genes. We select two largest classes from the liver dataset to do experiments.

The simulated data is created according to the protocol in [10], where the gene expression value is a normally generated random value with a noise generated from one uniform distribution of *U*(-0.1, 0.1), which is very similar to real data. In each simulated data, there are 1000 genes (first 50 with differential expression and next 950 with non-differential expression) and two classes *C*_1 _and *C*_2 _(having *n*_1 _and *n*_2 _samples, respectively). For any non-differential expression gene *j *(i.e. 51 ≤ *j *≤ 1000), its expression value *a*_*ij *_on each sample *i *is randomly generated from *N*(*μ*, 0.5) and *U*(-0.1, 0.1), where *μ *~*N*(0, 0.25). For gene *j *≤ 50, the value of gene *j *on any sample in class *C*_1 _is generated from *N*(*μ*_1_, *σ*_1_) and *U*(-0.1, 0.1), while that in class *C*_2 _is generated from *N*(*μ*_2_, *σ*_2_) and *U*(-0.1, 0.1), where *μ*_1_, *μ*_2_~*N*(0, 0.5). For the problem of multiple testing involved in identifying differential expression genes, bonfenorri correction of the significant level *α *can be used to reduce the error of type I. But a very small *α *will be disadvantaged to compare the performances of methods. In this paper, a relatively small significant level *α *will be used to control the type error I. On the real data, the value of *α *is set to 0.0001. On the simulated data, the significant level *α *is set to 0.01.

### Results on real data

**Figure 1:**
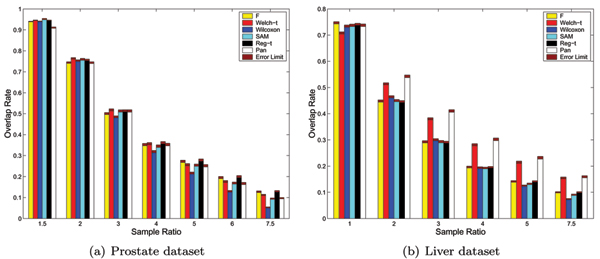
**The results on prostate and liver datasets under the evaluation model 1**. The expected Overlap Rates of six methods as well as their error limits on prostate and liver datasets under the evaluation model 1, where the sizes of samples in Class *C*_1 _of the artificial data, which are created from the liver data and the prostate data, are all fixed at 60.

**Figure 2:**
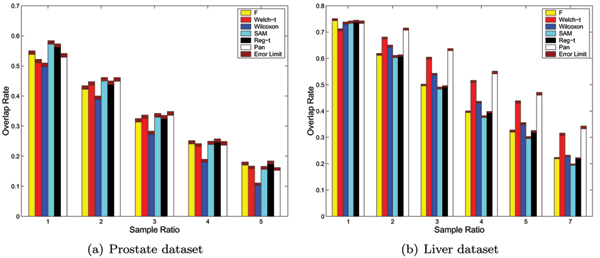
**The results on prostate and liver datasets under the evaluation model 2**. The expected Overlap Rates of six methods as well as their error limits on prostate and liver datasets under the evaluation model 2, where the number of overall samples in the artificial data from liver data is fixed at 120 and that from the prostate data is fixed at 60.

In the experiments of the evaluation model 1, the number of samples in Class *C*_1 _of the artificial data, which are created from the liver data or the prostate data, is always fixed at 60. The results under the evaluation model 1 are presented in figure 1. Because of the limitation of sample size in real data, in the experiments of the evaluation model 2, the value of *n*_1_+ *n*_2 _in the artificial data created from the liver data is fixed at 120 and that from the prostate data is fixed at 60. The results of the evaluation model 2 on real data are presented in figure 2. The expected Overlap Rates and its 0.95 confidence interval (or Error Limit) of each method at each specific SR are obtained from 100 randomly generated artificial data. Furthermore, in order to test whether the average Overlap Rate at *SR *≠ 1 (denoted as _*i*(*i *≠ 1)_)is significantly different with that at *SR *= 1 (denoted as _1_), we make a two sample t-test, where the observations are these 100 Overlap Rates calculated from 100 random artificial data with *SR *= 1 and those calculated from 100 random artificial data with *SR *≠ 1. So our null hypothesis states that _*i*(*i *≠ 1)_ = _1_, while the alternative hypothesis states that _*i*(*i *≠ 1) _≠ _1_. The p-values associated with the t-statistic in the evaluation model 1 and 2 are summarized in table 1 and 2, respectively. The experiments on real data indicate that the sample imbalance has a great influence on the performances of all six methods. As can be seen in figures 1 and 2, on both real datasets, the Overlap Rates of all methods are gradually decreasing in response to the increasing amounts of sample ratio. For example, in the figure 2(a), the margins between the average Overlap Rates at SR = 1 and that at SR = 3 on 6 methods (F, welch-t, wilcoxon, SAM, Regularized-t and Pan) are 0.2249, 0.1842, 0.2429, 0.2255, 0.2378 and 0.1932. According to the p-value showed in Table 2, we can conclude that on the real data the difference of the performance for each method between *SR *= 1 and *SR *≠ 1 has a very high statistical confidence. Additionally, there is also a difference among the Overlap Rates of different methods. It can be seen from figure 1 and 2 that Welch t-test and the method of pan create higher Overlap Rates on the unbalanced liver data than other 4 methods, while Wilcoxon test shows a lower Overlap Rate compared with other 5 methods on the unbalanced prostate data. However, because of without true solution, we can't decide directly and strictly which one of the six methods has the best performance on real data.

### Results on simulated data

In this section, under two proposed evaluation models, we generate two kinds of simulated data to compare the performances of different methods on the unbalanced data. In the first category, the differential expression genes have equal variances in sample class *C*_1 _and sample class *C*_2 _(i.e. *σ*_1 _= *σ*_2_), but have unequal variances (i.e. *σ*_1 _≠ *σ*_2_) in the second category of simulated data. The result on a simulated data is the average result on 1000 random data generated with a specific sample ratio.

#### Equal variances

**Figure 3:**
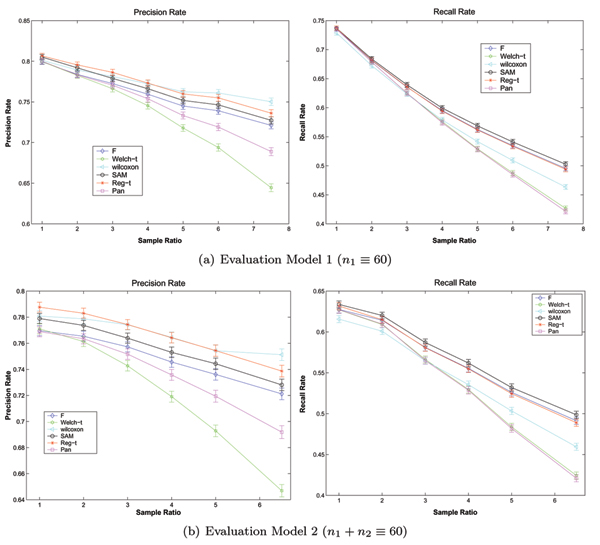
**The expected performances of six methods on the simulated data with equal variances, i.e. *σ*_1 _= *σ*_2_= 0.5**. The expected Precision Rates and Recall Rates of six methods as well as their error limits on the simulated data with equal variances (*σ*_1 _= *σ*_2 _= 0.5), where the number of samples of class *C*_1 _is fixed at 60 in the evaluation model 1 and the number of overall samples is fixed at 60 in the evaluation model 2.

Figure 3 shows the results on the simulated data in the case of equal variances (*σ*_1 _= *σ*_2 _= 0.5), where the number of samples in class *C*_1 _is fixed at 60 in the evaluation model 1 and the number of overall samples is fixed at 60 in the evaluation model 2. The corresponding p-values of the t-statistic on the simulated data with equal variances under the evaluation model 1 and 2 are summarized in table 3 and 4, respectively. From the experiments on simulated data with equal variances, we have the following:

The results on the simulated data with equal variances indicate the performances of all methods are greatly affected by the sample imbalance. Each of two metrics for the performance of method (Precision Rate and Recall Rate) is steadily declined as the sample ratio increases. This result is consistant with that of previous experiments on the real data.

Furthermore, the downward trend of Recall Rate in response to the increasing amounts of sample ratio is steeper than that of Precision Rate. In other words, the Recall Rate (the false negative) of the method for selecting differential expression genes is more sensitive than the Precision Rate (the false positive) to sample imbalance, although they are all affected by sample imbalance.

It is certain that the sample imbalance appears to have different effects between different methods. The difference between different methods become great when the degree of sample imbalance increases. In detail, the Precision Rates of the Wilcoxon rank-sum test and the Regularized t-test are higher than those of others, that is, the Wilcoxon rank-sum test and the Regularized t-test have lowest false positive rate (Type I error). Whereas, the Recall Rate of SAM is superior to that of other methods, i.e. the method of SAM has the lowest false negative rate (Type II error). And Welch t-test shows the worst performance.

#### Unequal variances

**Figure 4:**
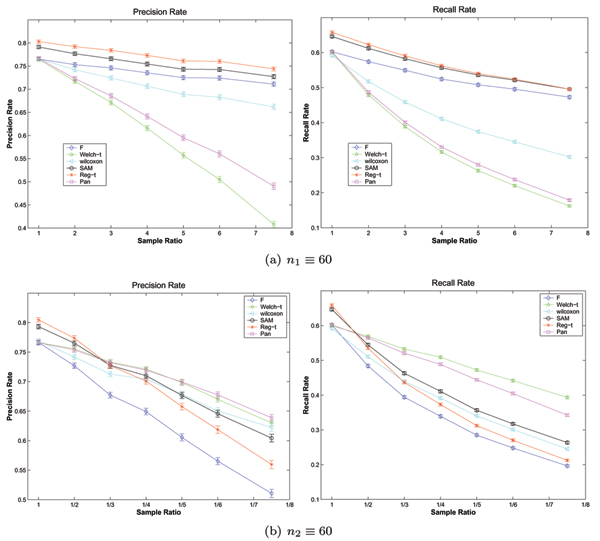
**The expected performances of six methods under the evaluation model 1 on the simulated data with unequal variances, where *σ*_1 _= 0.5, *σ*_2 _= 1**. The expected Precision Rates and Recall Rates of six methods as well as their error limits on the simulated data with unequal variances (*σ*_1 _= 0.5, *σ*_2 _= 1) in the evaluation model 1, where the numbers of samples of class *C*_1 _and class *C*_2 _are fixed at 60, respectively.

**Figure 5:**
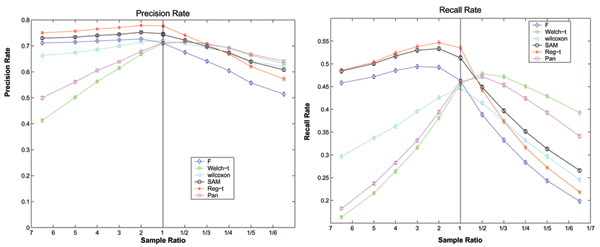
**The expected performances of six methods under the evaluation model 2 on the simulated data with unequal variances, where *σ*_1 _= 0.5, *σ*_2 _= 1 and *n*_1 _+ *n*_2 _≡ 60**. The expected Precision Rates and Recall Rates of six methods as well as their error limits on the simulated data with unequal variances (*σ*_1 _= 0.5,*σ*_2 _= 1) in the evaluation model 2, where the number of overall samples is fixed at 60.

In this section, under two evaluation models, the simulated data are generated in two case: the first case satisfies *σ*_1 _≤ *σ*_2 _and *n*_1 _≥ *n*_2_, for example, *σ*_1 _= 0.5, *σ*_2 _= 1 and *SR *= 1, 2, 3. The second case is that *σ*_1 _≤ *σ*_2 _and *n*_1 _≤ *n*_2_, for example, *σ*_1 _= 0.5, *σ*_2 _= 1 and *SR *= 1, , . The results of the evaluation model 1 on the two case of simulated data with unequal variances *σ*_1 _= 0.5, *σ*_2 _= 1 are showed in figure 4. Figure 5 plots the results of the evaluation model 2 with *n*_1 _+ *n*_2 _= 60 on two case of simulated data with unequal variances *σ*_1 _= 0.5, *σ*_2 _= 1.

As observed in figure 4 and 5, the performance of each of six methods degrades when the degree of sample imbalance increases, and on the same unbalanced data there exists great variance among the performances of six methods. These features are the same as those on the simulated data with equal variances. Furthermore, there are surprising variation on the performances of all methods compared in this paper between two different types of unbalanced data with unequal variances. In the case of *σ*_1 _= 0.5, *σ*_2 _= 1 and *n*_1 _≥ *n*_2_, Regularized t-test shows the highest Precision Rate and Recall Rate while Welch t-test performs the worst capability. In contrast, Regularized t-test has the medium performance and Welch t-test shows the best performance when *σ*_1 _= 0.5, *σ*_2 _= 1 and *n*_1 _≤ *n*_2_. This surprising observation can be easily explained by figure 5. As we can see in figure 5, the curve of each method performance under the evaluation model 2 is a function of sample ratio, which maximize its value at a specific sample ratio. These results imply that one should select a relatively feasible method to detect differentially expressed genes on an actual and specific unbalanced data. If one more suitable method has been selected to process the unbalanced data, then the result of analysis can be improved greatly.

**Figure 6:**
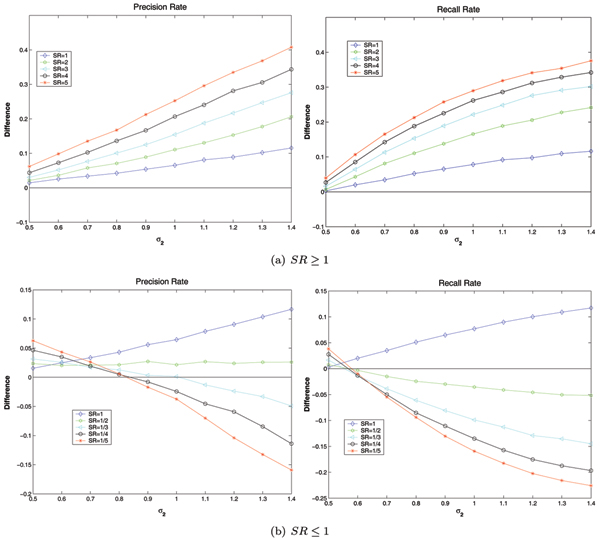
**The average performance Regularized t-test minus the corresponding performance of Welch t-test on the simulated data with varied variance *σ*_2_, where *n*_1 _+ *n*_2 _≡ 60 and *σ*_1 _≡ 0.5**. The average Precision Rate and Recall Rate of Regularized t-test minus that of Welch t-test on the simulated data with varied variance *σ*_2_, where *σ*_1 _≡ 0.5 and *n*_1 _+ *n*_2 _≡ 60.

In order to investigate the combined influence of sample ratio and varied variance on method performance, Regularized t-test and Welch t-test are selected as examples to demonstrate the dependency of the difference between methods with respect to different variances and sample ratios. Figure 6 shows the difference between Regularized t-test and Welch t-test against varied variance at different sample ratios. When *σ*_1 _≤ *σ*_2_, Regularized t-test is always superior to Welch t-test on the unbalanced data which satisfies *n*_1 _≥ *n*_2_. When *σ*_1 _≤ *σ*_2_and *n*_1 _≤ *n*_2_, the results become relatively complex. In the plot b of figure 6, several curves cross the line of zero, which implies that both methods of Regularized t-test and Welch t-test have some region of superiority. But when *σ*_1_≤ *σ*_2 _and *n*_1 _≤ *n*_2_, Welch t-test have obvious dominance. In addition, the more difference between variances *σ*_1 _and *σ*_2 _in unbalanced data, the higher different effects on different methods.

## Discussion

From this study, it is clear that there is a great effect on the performances of methods for selecting differential expression genes by the sample imbalance. Because of many objective factors, the gene expression data always involve the problem of small sample. As mentioned earlier in the previous section, coupled with the problem of small sample, the presence of the unbalanced data makes detecting differential expression genes more difficult. The sample imbalance is an important and inevitable problem in gene expression data analysis. Hence, one need to consider the problem of sample imbalance in the design of microarray experiments and the following data analysis.

Careful experimental design is necessary to improve the result of data analysis and reduce the cost of experiment simultaneously. By the comparison between plot a and b in figure 3, we can find that the expected Recall Rates and the expected Precision Rates at SR = 1 in plot b are higher than those at SR = 6 in plot a. In other words, because of the influence of sample imbalance, the result from one gene expression data of size 60 can be superior to that from another similar gene expression data of size 70. This finding is very considerable and exciting.

Furthermore, our results also indicate that on the unbalanced data, there have a great difference between the performances of different methods, especially on the data with heterogeneity. Some previous studies [24,25] have found that the variance  (for i = 1, 2) of expression values for gene *j *may depend on the mean expression value *μ*_*i*_. Hence, it will be very helpful to the result of analysis if a more suitable method has been selected to process the unbalanced data. For example, given an unbalanced data with unequal variances, one can improve the result of analysis if a feasible method from the six methods is selected. However, it is very likely that all six methods are not feasible for the unbalanced data and there is a requirement to find new methods more suitable to process the unbalanced data.

It should be noted that this paper does not consider the problem of determining sample size for detecting differentially expressed genes in microarray data. An interesting topic is how to assign samples between two groups in order to maximize a method performance under the constraint of the given number of overall samples *n*_1 _+ *n*_2_.

The results of this paper are based on six popular and typical methods for identifying differential expression genes including parametric method and nonparametric method. The similar effect of the sample imbalance on both kinds of methods leads us to believe that the findings in this paper should have, at least qualitatively, a comprehensive meaning. Also, two proposed evaluation models can be used to compare and evaluate other methods.

## Conclusion

The experiments in this paper demonstrate that sample imbalance has a great effect on identifying differential expression genes and two proposed models are effective to quantify the effect of sample imbalance. Moreover, different methods have different performances on the unbalanced data and we can not find one method to be suitable for all unbalanced data in the experiments. Among the six methods, the welch t-test appears to perform best when the size of samples in the large variance group is larger than that in the small one, While the Regularized t-test and SAM outperform others on the unbalanced data in other cases. In conclusion, two proposed evaluation models and the results provide some help in selecting suitable method to process the unbalanced data.

In future work, we will apply the evaluation models to evaluate more methods, for example the methods based False Discovery Rate. Furthermore, we attempt to investigate the problem of determining the sample size to maximize the performance of a given differential expression genes selection method.

## Methods

First, some notations used in this paper are introduced here. We assume there are *n *samples in the gene expression data and these *n *samples consist of two nonoverlapping categories named class one (*C*_1_) and class two (*C*_2_). In each sample, the expression values of *p *genes have been detected. Then the gene expression data may be represented by a *n *× *p *matrix

*A*_*n *× *p *_= (*a*_*ij*_)_*n *× *p*_,

where the element *a*_*ij *_is the expression value of gene *j *in sample *i*. The rows of *A *correspond to samples, and the *i-th *row vector of *A *is called the expression profile of the *i-th *sample. We assume that *n*_*k*_, _*k*_(*j*) and *S*_*k*_(*j*) are number of samples, sample mean and sample variance of gene *j *in the class *C*_*k*_, respectively, where *k *= 1, 2.

### Basic concepts

#### Definition 1 (Sample Ratio)

*Given a gene expression data, let n*_*k*_* denotes the number of samples in class C*_*k*_*, k = 1, 2. Then, Sample Ratio, denoted by SR, is defined to be n*_1_*/n*_2_*, i.e. SR = n*_1_*/n*_2_.

We use the Sample Ratio (SR) to measure the degree of sample imbalance between two groups. As revealed by definition 1, the further the value of SR departs from 1, the more serious the degree of sample imbalance is.

A key question also involved in this paper is how to evaluate the performance of a method for identifying differential expression genes, that is, how to evaluate the solution resulted from the method. For thousands of genes in a real gene expression data, it is generally unclear that which ones are differentially expressed genes. This situation has resulted in an obstacle to assess a method directly and strictly. In contrast, the true solution is known for the simulated data. So, in order to assess the performance of method directly, the simulated data are introduced. Furthermore, several measures are introduced to measure the quality of the method solution. Different measures are applicable in different situations, depending on whether a true solution is known or not.

First, we present a metric to assess the method performance for selecting differential expression genes on the real gene expression data.

Given real data, the whole real data is treated as the **original data (OD) **and the **artificial data (AD)**, which satisfies the given parameters *n*_1 _and *n*_2_, is generated by randomly sampling samples from the original data. Thus the *Overlap Rate *denoted by OR is calculated according to the following definition.

#### Definition 2 (Overlap Rate)

*Let DEG*_*OD*_* and DEG*_*AD*_* be the sets of Differentially Expressed Genes identified by some method on the original data (OD) and the artificial data (AD), respectively, then the Overlap Rate (OR) is defined as OR *= |*DEG*_*OD *_∩ *DEG*_*AD*_|/|*DEG*_*OD*_|.

To assess the method performance on the simulated data, we can compare the true solution with the suggested solution by the following method. Given simulated data with *p *genes, any solution can be represented by a binary 1 × *p *vector *T*, where *T(i) *= 1 if and only if the *i*-th gene is differentially expressed gene (or positive gene). Suppose that *T *and *S *be the true solution and the suggested solution of a method, respectively. And let *n*_*xy *_denote the number of pair (*i*, *i*), for which *T*(*i*) = *x *and *S*(*i*) = *y*, where *x, y *= 0 or 1. Thus, *n*_11 _is the number of true positive genes, *n*_01 _is the number of false positive genes, *n*_00 _is the number of true negative genes, and *n*_10 _is the number of false negative genes. Consequently, two different metricss, *Recall Rate *and *Precision Rate*, are introduced to measure the performance of method.

#### Definition 3 (Recall Rate)

*Suppose that S and T be the suggested solution of a differential expression gene selection method and the true solution, respectively. Then Recall Rate (RR) is defined as RR *= *n_11_/*(*n*_10 _+ *n*_11_).

#### Definition 4 (Precision Rate)

*Suppose that S and T be the suggested solution of a differential expression gene selection method and the true solution, then Precision Rate (PR) is defined as PR = n*_11_/(*n*_01_+*n*_11_).

From the definitions 3 and 4, We can see that the Recall Rate focuses on the false negative while the Precision Rate focuses on the false positive. However, the false negative and the false positive are two different keystones in the context of selecting differential expression genes, and the false negative is inconsistent with the false positive. So in a particular problem specification, one can choose either Recall Rate or Precision Rate as the main focus.

### Random sampling

For one specific method of differential expression genes selection and one given data with *n*_1 _samples in class *C*_1 _and *n*_2 _samples in class *C*_2_, we can only get one specific value of each of the metrics OR, RR and PR of the method on the given data. So after one specific method and the set of gene expression data with size *m* are given, there exists the set of ORs (RRs or PRs) with size *m* resulted from the method. A perfect way to evaluate the performance of one method is to run the method on the whole set of gene expression data which satisfy given parameters *n*_1 _and *n*_2 _and to calculate the average value of each metric. But the cardinality of the set of data with parameters *n*_1 _and *n*_2 _may be very large or infinite. For example, if a real microarray data has 50 and 30 samples in class *C*_1 _and *C*_2_respectively, then the number of different artificial data with parameters *n*_1 _= 40 and *n*_2 _= 20 is  > 3 × 10^17^. In order to reduce the computation cost and avoid the problem of infinity, one feasible way is to estimate the expected value of each metric and its approximate confidence interval (or Error Limit) by sampling a sample from the specific gene expression data randomly.

#### Lemma 1

[28]*Suppose that population X has mean μ, and finite variance σ*^2^, *and X*_1_, *X*_2_*, ..., X*_*n *_*are an independent random sample of size n from the population X, then the sample mean **is an unbiased estimate of μ and the sample variance S*^2^*is an unbiased estimate of σ*^2^. *Moreover, the variance of *, *denoted by D*(), *satisfies D*() = *σ*^2^/*n*, *where*



According to lemma 1, we can use the sample mean  to estimate the population mean *μ *and calculate its approximate confidence interval. In sampling survey, the exact distribution of the estimate (i.e. ), is unknown. However, according to the central limit theorem, we can expect the sampling distribution of  to be approximately normal distribution with mean *E*() = *μ *and variance *D*(). That is



As a result, a (l-*α*)100% approximate confidence interval for the estimate of *μ *is . In practice, the standard deviation of sampled population *σ *is typically unknown. Replacing *σ *by *S *leads to the corresponding estimate  and  is referred to as the standard error (SE) of . Therefore, a feasible confidence interval of *μ *at a significant level *α *is [ - *z_α_*,  + *z_α_*] and the approximate Error Limit (EL) is *z_α_*. For sample of size *n *≥ 30, regardless the shape of most population, sampling theory guarantees good results [28].

When population is finite, the change is the introduction of the factor 1 - *f *for the variance *D*(), where *f *= *n/N *is the sampling fraction and *N *is the size of population. The factor 1 - *f *is called the finite population correction (fpc). That is, the confidence interval is . In practice, the fpc can be ignored whenever the sampling fraction does not exceed 5% [29].

### Evaluation models

In order to investigate the effect of sample imbalance on differential expression genes selection, one simply needs to consider the change of the performance of a method in response to different sample ratios (SRs), because the sample ratio is a measure of the degree of sample imbalance between two groups. Therefore, two evaluation models are proposed as follows.

#### Evaluation model 1

*Let the number of samples of certain class always equal to constant C, for instance n*_1_*= C, and the artificial data (or the simulated data) is randomly created with different Sample Ratios. Then compare the method results on the data with various Sample Ratios*.

#### Evaluation model 2

*Let the number of all samples in the artificial data (or the simulated data) always equal to constant C, i.e. n*_1_+*n*_2 _≡* C, and the artificial data (or the simulated data) is randomly created with different Sample Ratios. Then the method is evaluated based on these random data with particular parameter SR*.

### Calculating cutoff point

For the parametric method, the cutoff point of a significance level *a *is calculated from the assumed distribution. In the nonparametric method, for a given significance level *α*, following the spirit of SAM, we find the 100(1 - *α*)% quantile of the null distribution, i.e. noted as , using the following formula



where B is the number of permutations and  is the value of the statistic for the *i*-th gene in the b-th permutation. Then the quantile value  is used as the cutoff point for that statistic to select differential expression genes.

## Authors' contributions

K.Y. conceived the study, performed the implementations and drafted the manuscript. H.G. critically read and revised the final manuscript. J.L. supervised the whole work and finalized the manuscript. All authors read and approved the final manuscript.

## Figures and Tables

**Table 1:** The p-value of t-statistic under the evaluation model 1 on two real datasets.

SR	2	3	4	5	6	7.5
Prostate						
F	1.4e-115	l.0e-162	9.9e-174	1.8e-188	1.6e-201	6.2e-232
welch-t	2.3e-112	3.3e-157	5.0e-177	4.4e-189	3.4e-213	3.2e-249
sam	4.8e-090	1.3e-146	3.0e-164	3.1e-186	7.9e-203	4.8e-230
wilcoxon	1.3e-112	1.4e-156	6.3e-182	9.5e-202	1.6e-230	1.2e-279
Reg-t	2.8e-108	4.9e-159	3.9e-170	1.3e-183	9.7e-199	2.8e-229
Pan	1.7e-084	2.8e-135	1.3e-154	5.9e-176	2.0e-192	9.3e-227

Liver						
F	3.1e-118	5.3e-151	4.6e-171	4.7e-188	7.3e-198	1.5e-204
welch-t	9.8e-086	1.9e-122	1.8e-144	1.7e-156	6.6e-173	7.8e-185
sam	6.1e-106	3.5e-139	7.4e-166	2.0e-178	2.8e-187	3.1e-195
wilcoxon	2.6e-107	1.3e-148	1.6e-173	1.7e-189	1.2e-200	2.9e-211
Reg-t	3.4e-119	4.7e-153	8.9e-177	1.2e-188	8.6e-198	8.8e-205
Pan	5.1e-073	1.1e-111	7.0e-135	1.8e-144	5.9e-165	1.8e-177

**Table 2:** The p-value of t-statistic under the evaluation model 2 on two real datasets.

SR	2	3	4	5	7
Prostate					
F	7.2e-030	9.0e-068	8.8e-089	7.1e-107	
welch-t	1.8e-016	6.9e-056	5.6e-087	1.6e-110	
sam	9.2e-034	4.7e-071	1.6e-099	9.0e-121	
wilcoxon	1.6e-028	3.7e-070	5.2e-099	2.6e-123	
Reg-t	1.2e-033	1.1e-073	1.3e-094	1.0e-113	
Pan	2.1e-017	2.0e-054	5.5e-083	2.8e-112	

Liver					
F	1.6e-060	8.9e-111	1.2e-137	6.5e-147	1.6e-177
welch-t	3.2e-008	4.5e-049	1.8e-085	5.9e-106	1.6e-137
sam	1.3e-050	5.5e-099	1.1e-129	9.5e-142	2.0e-170
wilcoxon	3.4e-036	3.1e-089	3.1e-121	5.0e-136	8.9e-171
Reg-t	6.3e-062	1.4e-112	6.5e-138	8.6e-148	2.1e-178
Pan	3.1e-005	1.7e-039	1.3e-069	1.2e-094	6.5e-124

**Table 3:** The p-Value of t-statistic on the simulated data with *σ*_1 _= *σ*_2 _= 0.5, under the evaluation model 1 (*n*_1 _≡ 60).

SR	2	3	4	5	6	7.5
Precision						
F	2.1e-10	5.8e-20	3.5e-50	3.0e-085	2.1e-096	7.6e-150
welch-t	2.3e-12	1.9e-36	9.3e-85	3.8e-164	4.2e-231	0.0
sam	5.9e-08	4.5e-24	2.6e-47	3.1e-079	3.3e-092	1.7e-144
wilcoxon	1.5e-06	1.7e-14	2.3e-26	1.4e-044	9.5e-045	3.3e-064
Reg-t	1.7e-06	2.7e-15	3.2e-36	1.6e-063	3.6e-073	4.2e-123
Pan	6.7e-12	5.3e-29	1.9e-61	6.8e-117	5.5e-152	1.0e-228

Recall						
F	6.6e-76	2.0e-211	0	0	0	0
welch-t	2.1e-81	4.1e-247	0	0	0	0
sam	3.7e-74	8.5e-204	0	0	0	0
wilcoxon	9.0e-80	1.8e-228	0	0	0	0
Reg-t	3.4e-79	5.9e-215	0	0	0	0
Pan	3.1e-82	8.3e-246	0	0	0	0

**Table 4:** The p-Value of t-statistic on the simulated data with *σ*_1 _= *σ*_2 _= 0.5, under the evaluation model 2 (*n*_1 _+ *n*_2 _≡ 60).

SR	2	3	4	5	6.5
Precision					
F	6.6e-2	5.3e-06	5.9e-17	4.2e-029	3.4e-055
welch-t	2.5e-4	3.7e-23	1.6e-44	7.1e-128	2.3e-254
sam	3.1e-2	4.0e-08	1.6e-19	1.0e-030	7.3e-061
wilcoxon	2.3e-l	1.1e-02	1.2e-08	1.2e-018	6.3e-022
Reg-t	4.6e-2	6.6e-07	8.7e-17	4.0e-029	1.4e-056
Pan	2.6e-2	1.1e-09	1.5e-29	1.8e-056	1.4e-112

Recall					
F	9.2e-6	2.3e-47	6.6e-102	1.0e-180	1.2e-288
welch-t	2.0e-9	6.2e-77	1.4e-169	1.9e-309	0
sam	3.7e-6	1.0e-48	7.9e-103	3.7e-184	1.9e-290
wilcoxon	7.2e-7	5.2e-56	3.5e-122	2.3e-213	0
Reg-t	2.0e-8	4.7e-57	2.6e-115	3.1e-199	2.9e-310
Pan	4.2e-9	1.7e-80	4.6e-172	8.1e-315	0
